# Key population-led community-based same-day antiretroviral therapy (CB-SDART) initiation hub in Bangkok, Thailand: a protocol for a hybrid type 3 implementation trial

**DOI:** 10.1186/s43058-022-00352-9

**Published:** 2022-10-01

**Authors:** Sita Lujintanon, Sorawit Amatavete, Supanat Thitipatarakorn, Thanyawee Puthanakit, Wipaporn Natalie Songtaweesin, Tanachai Chaisalee, Surang Janyam, Nittaya Phanuphak, Reshmie A. Ramautarsing

**Affiliations:** 1 Department of Epidemiology, Johns Hopkins Bloomberg School of Public Health, 615 N Wolfe Street, Baltimore, MD 21205 USA; 2 Institute of HIV Research and Innovation (IHRI), Bangkok, Thailand; 3 Division of Infectious Diseases, Department of Pediatrics, Faculty of Medicine, Chulalongkorn University (PIDCU), Bangkok, Thailand; 4 Rainbow Sky Association of Thailand (RSAT), Bangkok, Thailand; 5 Service Workers In Group Foundation (SWING), Bangkok, Thailand

**Keywords:** HIV, Antiretroviral therapy initiation, Community-based service, Men who have sex with men, Transgender women, Key population, Telehealth, Implementation research

## Abstract

**Background:**

Same-day antiretroviral therapy (SDART) initiation, in which people living with HIV (PLHIV) who are antiretroviral therapy (ART)-naïve, willing, and clinically eligible start ART on the same day of HIV diagnosis, has been implemented in several healthcare facilities in Thailand since 2017. This evidence-based practice has demonstrated increased ART uptake, virologic suppression, and retention in care. However, linkage to care gaps exist in community-based organizations (CBOs) in Bangkok whereby as much as 20% of key populations (KP), mainly men who have sex with men and transgender women, living with HIV were lost to follow-up pre-ART initiation. To increase access to and uptake of ART among these populations, this study proposes that trained KP lay providers should lead community-based ART (CB-SDART) initiation service. This protocol describes the combined use of the Proctor’s implementation outcome framework and the Consolidated Framework for Implementation Research to guide and evaluate the CB-SDART implementation.

**Methods:**

This study follows the hybrid design type 3: it is an implementation trial that secondarily assesses service and client outcomes by comparative interrupted time series analysis. Five strategies have been formulated to meet three implementation outcomes (i.e., feasibility, fidelity, and sustainability): (1) developing stakeholder relationships by engaging the CBO leaderships, (2) training and educating KP lay providers, (3) adapting and tailoring SDART to CBO-specific context, (4) using evaluative and iterative strategies to assess adherence to standard operating procedures, and (5) developing stakeholder relationships by engaging external stakeholders. Teleconsultation with physicians and ART home delivery will be integrated as another ART initiation option for clients and allow service provision during the COVID-19 pandemic. A mixed-method assessment will be conducted on key stakeholders and PLHIV diagnosed at two implementing CBOs, Rainbow Sky Association of Thailand and Service Workers in Group Foundation, in Bangkok, Thailand.

**Discussion:**

This implementation research may be the first to provide robust data at the implementation, service, and client levels to inform how to successfully task-shift SDART initiation service to trained KP lay providers and facilitate the expansion of CB-SDART in the future.

**Trial registration:**

This trial was registered with the Thai Clinical Trial Registry as TCTR20210709004 on July 9, 2021.

**Supplementary Information:**

The online version contains supplementary material available at 10.1186/s43058-022-00352-9.

## Contributions to the literature

- This study is among the first in Thailand to demonstrate *how* implementation frameworks are used to inform study designs and strategies to generate robust evidence at the implementation, service, and individual levels on *how* to effectively implement the same-day antiretroviral therapy (SDART) initiation service in a community setting.
- SDART is led by trained key population lay providers and incorporates telehealth technology, making it suitable for key populations living with HIV and the COVID-19 pandemic period.
- This innovative approach is expected to serve as a service model to accelerate linkage to care and expand the lay providers’ role in HIV care.

## Background

Same-day antiretroviral therapy (SDART) initiation is an evidence-based practice (EBP) that has expedited linkage to care in healthcare facilities across the globe [1–5] and it is recommended by national and international guidelines [6, 7]. This EBP has proven to be efficient, with increased antiretroviral therapy (ART) uptake [2, 4], virologic suppression, and retention in care [1–4, 8]. While Thailand has implemented SDART initiation in several healthcare facilities since 2017 [4, 5, 9], only 80% of Thai people living with HIV were on ART [10], with an even lower percentage of 62% in Bangkok in 2019 [11]. Linkage to care gaps particularly exists in community settings. As much as 20% of key populations, particularly men who have sex with men and transgender women, who tested HIV positive at community-based organizations (CBOs) in Bangkok, namely Rainbow Sky Association of Thailand (RSAT) and Service Workers in Group Foundation (SWING), were lost to follow-up (LTFU) before they were successfully referred for facility-based ART initiation. In addition, timely ART initiation on the same day of HIV diagnosis has been a challenge due to the logistical and geographical barriers of the referral process [4]. Nonetheless, RSAT and SWING have the strengths in providing friendly and stigma-free services under the key population-led health service (KPLHS) model [12, 13] and in index case finding in which they accounted for 6.8 and 5.1% of new HIV diagnoses in Bangkok, respectively, in 2019 [14]. A task-shifted, community-based approach in which the trained key population lay providers lead ART initiation under the supervision of physicians is a novel solution. While the effectiveness data of community-based ART initiation is emerging [15], most evidence of SDART implementation pertains to clinical outcomes [1–5, 9, 16–19], and evidence on implementation fidelity and intervention adoption variables that may influence the effects of SDART is lacking. Therefore, we designed a study to inform how to implement the community-based SDART (CB-SDART) initiation hubs at RSAT and SWING in order to improve acceptability and access to, increase uptake of, and overall accelerate ART initiation among key population living with HIV.

## Methods

### Study aims

The aims of this study protocol are to document and inform how to implement SDART in the community setting, and to assess the feasibility, fidelity, and sustainability of CB-SDART by describing and explaining the processes and factors influencing these implementation outcomes (IOs). Additionally, we will assess service and client outcomes, including timeliness, patient centeredness, function, and symptomatology of, and satisfaction with CB-SDART, as a secondary objective.

### Study design

The study design is informed by the Proctor’s implementation outcome framework [20] and the Consolidated Framework for Implementation Research (CFIR) [21]. The Proctor’s implementation outcome framework, which clearly sets apart IOs from service and client outcomes, is used to determine the relevant IOs for the evaluation of the implementation process [20]. CFIR, which is a determinant framework, is used to assess the contextual domains of importance in the implementation of CB-SDART, as well as constructs within those CFIR domains, to identify factors of influence and potential barriers and facilitators [21]. This combined framework of Proctor’s implementation outcome framework and CFIR yields a set of implementation strategies (ISs) that will guide the implementation and evaluation of CB-SDART.

This resulted in an implementation trial that follows the hybrid design type 3 in which the primary outcomes are IOs and the study secondarily observes and collects data on the service and client outcomes of CB-SDART [22], using mixed-method documentation and evaluation. This includes the use of in-depth interviews, record archives, electronic case report form (eCRF), and checklists.

**Fig. 1:**
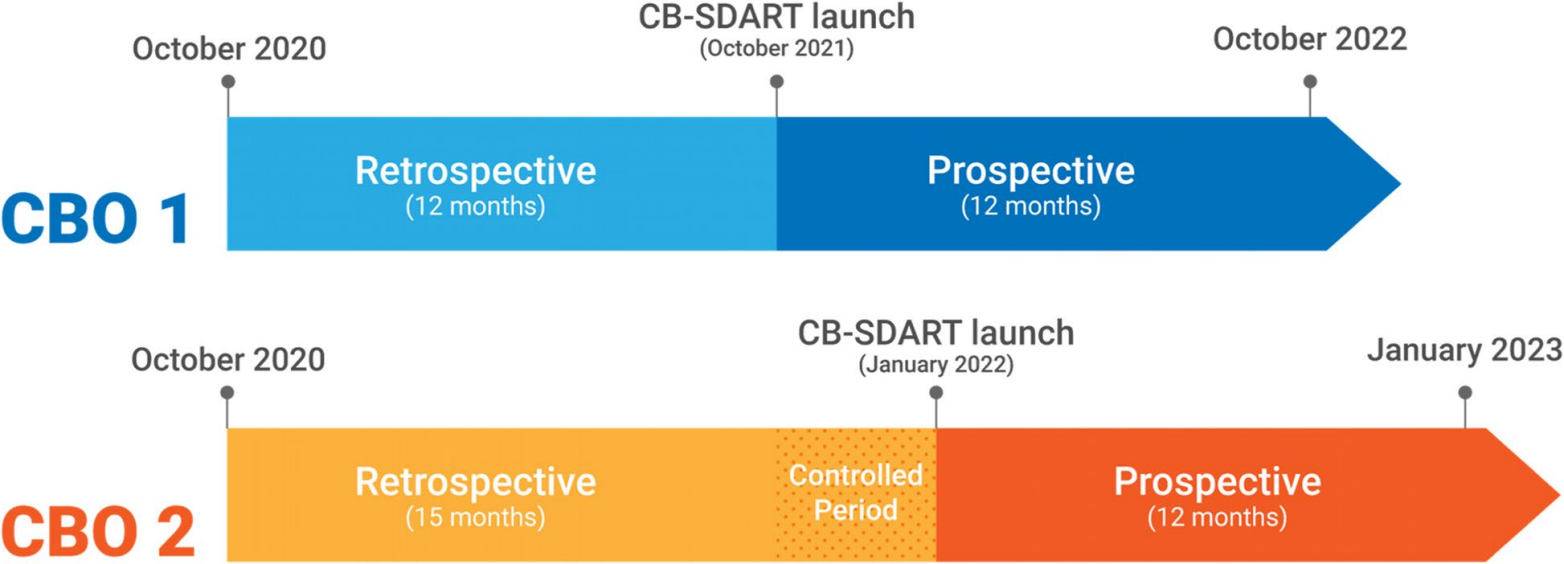
Community-based same-day antiretroviral therapy delivery timeline according to comparative interrupted time series study design

To assess the secondary service and client outcomes, a comparative interrupted time series (CITS) design will be used. In this study, a time series of outcomes of interest is used to establish an underlying trend, which is interrupted by the implementation of CB-SDART [23]. The counterfactual scenario in which CB-SDART has not been implemented and in which the trend continues unchanged provides a comparison for the evaluation of the impact of the intervention by examining any changes occurring in the post-intervention period. By launching CB-SDART at one CBO first while the other CBO will serve as the comparison group for a period of 3 months before the intervention will be launched at the second CBO, this allowed additional comparison and takes into account time-varying confounders. The timeline for CB-SDART launch is depicted in Fig. 1. The study timeline can be found in Additional file 1. The total study duration is approximately 2 years with the implementation phase starting in October 2021. A self-administered survey will also be conducted among clients to assess satisfaction with CB-SDART.

This protocol is reported according to the Standard Protocol Items: Recommendations for Intervention Trials (SPIRIT; Additional file 2) [24, 25].

### Study setting

The study will be conducted at 2 CBOs in Bangkok: RSAT and SWING. RSAT is situated in Ramkhamhaeng, a highly populated suburban area of Bangkok with Thailand’s largest public university and any auxiliary services deemed necessary for living. SWING has 2 locations with one located in the middle of Patpong, Bangkok’s oldest red-light district, and another located in Saphan Kwai, a thriving commercial and residential area with convenient transportation routes. Both CBOs provide sexual health services under the KPLHS model, including HIV and sexually transmitted infection (STI) testing and counseling, and pre-exposure prophylaxis (PrEP) and post-exposure prophylaxis (PEP) distribution for key population communities [12, 13].

### Study population

The study population includes CB-SDART key stakeholders and clients. The key stakeholders include key population lay providers, CBO leaderships, and physicians involved in CB-SDART delivery. No sample size calculations will be performed for the IO assessment, and purposive sampling will be conducted to screen participants for the mixed-method assessment until data saturation is reached. However, in-depth interviews will be conducted to collect qualitative information on a subset of 10 key population lay providers and CBO leaderships at each CBO during the pre-implementation phase and at months 6 and 12, or until saturation is reached [26], to assess feasibility and sustainability. During the implementation period, all clients who test HIV positive at CBOs, are Thai nationals, are at least 13 years, the minimum Thai legal age of consent to receive sexual health services, and have never received ART are eligible and will be offered CB-SDART. The clients from the pre-CB-SDART launch period with the same eligibility criteria will be used as a comparison group. A sample size calculation to compare two independence population proportion with *α* = 0.05 and power = 80% was conducted to ensure an adequate sample size for the detection of at least 20% difference in ART initiation rate between the pre- and post-CB-SDART launch periods and assumed 10% LTFU rate. At least 160 HIV-positive participants from the two CBOs must be enrolled from each period. After meeting the sample size, the clients will continue to be enrolled into the study for a period of 1 year as we work to achieve feasibility, fidelity, and sustainability of CB-SDART implementation. Additionally, 10 clients at each CBO at months 3 and 9 will participate in an in-depth interview or until saturation is reached to assess service satisfaction.

### Intervention

The CB-SDART initiation hub model allows ART-naïve people living with HIV who are willing and clinically ready to start ART on the same day of HIV diagnosis or confirmatory test at the CBOs. CB-SDART has been adapted from the SDART initiation hub model at the Thai Red Cross Anonymous Clinic (TRCAC), implemented by the same study team [4, 5, 9, 16–19]. The core components of SDART as shown in Table 1 remain the same, while the periphery components are modified to suit the community setting. This includes that CB-SDART will be run almost entirely by trained key population lay providers under the supervision of non-specialist and/or infectious disease physicians. Since the COVID-19 pandemic, telehealth has been successfully incorporated into the SDART service flow at TRCAC for follow-up visits, with medical consultation via video call and ART refill by home delivery [16–18]. This practice will be extended to the ART initiation visit whereby on-call physicians may conduct virtual physical examination and symptomatic screening to assess opportunistic infections (OIs) and clinical complications in order to assist key population lay providers in complicated and/or requested cases.

**Table 1:** Core and periphery components, procedures, and visits of same-day antiretroviral therapy

SDART core components	Adapted periphery components	SDART procedures	Timepoint			
			**Initiation visit: day 0**	**Follow-up visit: day 14**	**Refer-out: day 45**	**Long-term follow-up: day 46–day 730**
Interactive counseling to assist clients to make an informed decision regarding ART initiation and lifelong adherence as well as to set positive treatment and self-care goals in order to achieve viral suppression	Increasing the role of KP lay provider in leading ART initiation service in addition to their case management and support role; adding option to return to CBO for point-of-care viral load testing at 6 months after ART initiation	CB-SDART eligibility assessment	✓			
		Psychosocial readiness and willingness assessment	✓			
		Ongoing counseling and adherence support provided by peer navigators	✓	✓	✓	✓
Screening to rule out serious opportunistic infections before ART initiation	Chest X-ray and selected laboratory testing conducted outside of the clinic; adding point-of-care CD4 count testing to aid ART initiation and physical examination led by KP lay providers under the remote supervision of physicians	Symptomatic screening	✓			
		Sample collection for baseline laboratory testing, STI sample collection (if accepted)	✓			
		Chest X-ray	✓			
		Informing baseline laboratory test results		✓		
ART initiation within the same day of HIV diagnosis for clients who are ready and willing to start ART without clinical complications, if possible	Adding ART initiation and follow-up options, including in-person at the CBOs or via telehealth, with KP lay providers or physicians; adding ART home delivery option	ART prescription	✓			
		Management of adverse drug events		✓		
		ART modification (if required) and refill		✓		
Referral system at the ready to assist clients to change their facility coverage according to their national health insurance and in referral process	KP lay providers assisting clients in navigating the health system and in the referral process	Refer for OI investigation (for clinically ineligible clients)	✓			
		Change in facility coverage to utilize the national health insurance (if requested)	✓			
		Warm-hand off (if requested)			✓	

*SDART* Same-day antiretroviral therapy, *CB-SDART* Community-based same-day antiretroviral therapy, *ART* Antiretroviral therapy, *CBO* Community-based organization, *STI* Sexually transmitted infection, *KP* Key population, *OI* Opportunistic infection

The CB-SDART procedures will be as follows: After reactive result was confirmed by three different anti-HIV tests (4th generation Alere™ HIV Combo rapid test, SD Bioline HIV 1/2 3.0, Rapid Test for Antibody to HIV (Colloidal Gold Device)), HIV-positive clients will be assessed for CB-SDART eligibility criteria by a trained key population lay provider. Eligible clients will be offered to join the CB-SDART study and undergo the informed consent process. This includes assessing for their willingness to start ART at the CBOs, and by which method, including in-person or through telehealth (i.e., clinical consultation via video call and ART home delivery) integrated ART initiation led by key population lay providers or physicians. Those who decline CB-SDART, including those who decline treatment and those who want to start ART elsewhere, will be referred to an appropriate facility with the assistance of the key population lay providers. Those who accept CB-SDART will undergo pre-ART initiation counseling and sample collection for baseline laboratory tests. The sample will be processed at the CBOs for the following labs: CD4 cell count, syphilis serology (rapid plasma reagin (RPR) and Treponema pallidum particle hemagglutination (TPHA)), hepatitis C antibody (anti-HCV), and urine analysis, with a total approximate turnaround time of 1 hour. The sample will be processed at an outsourced laboratory for the following labs: alanine aminotransferase (ALT) test, creatinine/creatinine clearance, and hepatitis B surface antigen (HBsAg) test, with a total of approximate turnaround time of 24 hours. Clients will travel to a nearby hospital to receive a chest X-ray. Cryptococcal antigen will be tested only for those with CD4 count < 100 cells/mm^3^. Point-of-care rapid testing for chlamydia (CT) and gonorrhea (NG) will be offered to all clients. Only anti-HIV and chest X-ray results will be required for ART initiation; point-of-care CD4 count will be considered to facilitate ART initiation in the community setting. Clients will receive pre-ART counseling, including psychosocial readiness assessment using depression screening (2Q and 9Q) [27] and Life-Steps adherence counseling. Tuberculosis (TB), cryptococcal meningitis, and other serious opportunistic infections/illnesses will be ruled out by using a symptomatic screening checklist and conducting a focused physical examination. Clinically ineligible clients will be referred to their preferred facility for diagnosis, treatment, and/or ART initiation. For clinically eligible clients, 1 month of the first-line ART regimen according to the current version of the Thailand National Guidelines on HIV/AIDS Diagnosis, Treatment, and Prevention will be prescribed. The current 2020/2021 guidelines recommend tenofovir/lamivudine/dolutegravir or tenofovir/emtricitabine/dolutegravir [28]. After ART initiation, clients diagnosed or suspected with STIs, hepatitis C, and other health conditions will be referred for confirmed diagnosis and/or treatment. To prepare for referral to a long-term HIV treatment facility, a key population lay provider will assist the clients in the change in facility coverage process in order to utilize the national health insurance at a convenient and/or available facility. Clients will be provided contact details of a key population lay provider they can reach if they experience suspected adverse events (AEs) and/or require additional consultation. Clients who experience AEs, such as adverse drug reactions and allergic reactions, will be informed to contact a team of physicians and key population lay providers immediately to assess the severity and create a proper management plan.

For the follow-up visit at week 2, the clients have the options to follow up in-person at the CBOs or via telehealth with the key population lay providers or physicians. Clients who do not have access to a device for making video call will have the option to follow up via voice call and sending photos. At the follow-up visit, the clients will be assessed for AEs and those who tolerate the ART well will receive another 1-month supply of ART to bridge the transition to their long-term HIV treatment facility. A key population lay provider will also accompany the clients to their long-term facility upon request to ensure successful linkage to long-term care. All clients who accept CB-SDART will receive a follow-up support for at least 1 year at months 3, 6, and 12 from a team of key population lay providers who will also assess their health outcomes, including virologic suppression, which is defined as < 50 copies/mL. The clients will be offered to come back for an optional follow-up at the CBO at 6 months after ART initiation for viral load, syphilis, CT, NG, HBsAg, and anti-HCV testing. Eligible clients who decline CB-SDART will be followed up until they start treatment and the following 1 year upon their consent. All ART initiation and follow-up services are covered by the study, except for the cost from referral for confirmed diagnosis and/or treatment and long-term HIV treatment in which the clients have the option of receiving free services covered by the national health insurance.

The intervention is reported according to the Template for Intervention Description and Replication (TIDieR; Additional file 3) [29].

### Implementation outcomes and strategies

Three relevant Proctor’s IOs were selected: feasibility, fidelity, and sustainability. Feasibility is defined as the ability of trained key population lay providers to lead and provide SDART and the adaptability of SDART initiation hub model to the community setting. Fidelity is defined as the degree to which core components of SDART as shown in Table 1 are delivered as intended. Sustainability is defined as internal sustainability in which the CBO leaderships commit to CB-SDART implementation and demands for sustainment, and external sustainability in which national public health agencies, particularly National Health Security Office (NHSO) and Bangkok Metropolitan Administration (BMA), recognize and endorse the implementation of CB-SDART.

**Fig. 2:**
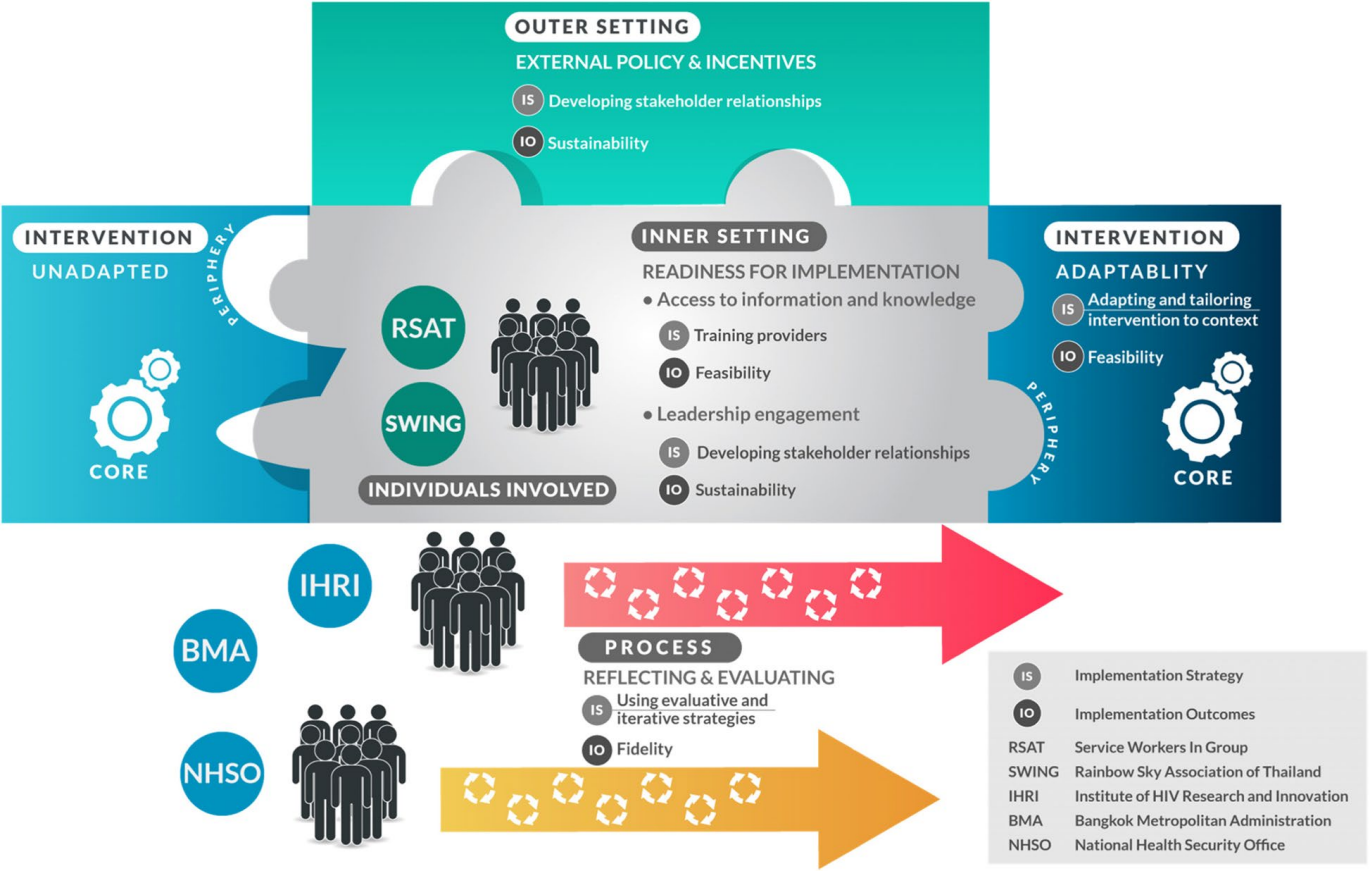
Implementation framework developed by identifying Proctor’s implementation outcomes and implementation strategies through the assessment of Consolidated Framework for Implementation Research’s constructs

In order to formulate ISs to achieve these IOs, a community engagement and consultation meeting was held between RSAT and SWING leaderships, key population lay providers, and IHRI implementers. During the meeting, both CBOs emphasized the importance of adaptability of SDART procedures to suit the CBO setting, including using point-of-care CD4 testing, which is available at the CBOs, to assist ART initiation via telehealth, and providing intensive training for the key population lay providers to lead physical examination and symptomatic screening. The issue of sustainability of KPLHS model was also raised, which the CBOs proposed should be achieved through NHSO’s financial support for CBOs to provide HIV services across Reach-Recruit-Test-Treat-Prevent-Retain cascade. The CBOs’ input and concerns were mapped onto the CFIR to facilitate the planning of ISs. As a result, 4 CFIR domains (intervention, inner setting, outer setting, and process) were identified and deemed essential for the implementation success in order to meet the three chosen Proctor’s IOs (Fig. 2) [20, 21].

From the CFIR domain “intervention characteristic,” we will focus on “adaptability” to meet the IO “feasibility.” Feasibility refers to the degree to which an intervention can be adapted and tailored to meet the local needs. An intervention that can be easily modified to adapt to each setting is more likely to achieve implementation success [30, 31]. Hence, collaborative efforts between IHRI, PIDCU, and CBOs in standard operating procedure (SOP) development will be undertaken to adapt and tailor the periphery components of SDART to the CBO-specific inner and outer settings in order to increase intervention feasibility while the core components remain intact.

From the CFIR domain “outer setting,” we will focus on “external policy and incentives” to meet the IO “sustainability,” specifically external sustainability. This construct is broad and includes different external strategies to spread interventions. Political directives have shown to increase motivation of organizations to implement interventions [32]. Throughout the process of implementation, relationships with public stakeholders, particularly NHSO and BMA, will be built and maintained through regular reporting and meeting in order to gain their recognition and endorsement, which are imperative for sustainability.

From the CFIR domain “inner setting,” we will focus on “readiness for implementation.” This is reflected in tangible and immediate indicators showing an organization’s commitment to its decision to implement an intervention. Two sub-constructs of particular importance that contribute to the organization’s readiness for implementation are “leadership engagement” and “access to information and knowledge.” “Leadership engagement” will address the IO “sustainability,” specifically internal sustainability. Therefore, engagement with the CBO leaderships throughout the decision-making processes will be ensured to develop the sense of ownership and the demand for the sustainment of SDART at CBOs. “Access to information and knowledge” on the other hand will facilitate the IO “feasibility.” Easy access to information and knowledge is essential for successful implementation. Therefore, providing didactic and skill training, and continuous coaching and mentoring to educate and build the capacity of key population lay providers to lead the implementation of SDART will be ensured. Since the key population lay providers have already undergone training and certification in sexual health counseling, HIV and STI testing, and distribution of STI treatment, PrEP, PEP, and ART, additional training will be provided for CB-SDART delivery.

From the CFIR domain “process,” all four constructs (i.e., planning, engaging, executing, and reflecting and evaluating) will be done incrementally and simultaneously, and each activity will be revisited, expanded, refined, and re-evaluated throughout the implementation process. Special focus will lie on “reflecting and evaluating” to meet the IO “fidelity,” as processes of reflection will help foster a learning climate within and between each participating organization, through which odds for future implementation will be improved by the uncovering of root causes of successes and failures [21]. Iterative reflection and evaluation will be conducted continuously and internally, led by the CBO leaderships with the support of IHRI, to ensure intervention fidelity.

Ultimately, based on key stakeholder input, five ISs were formulated to ensure implementation success:

1. Developing stakeholder relationships by engaging the CBO leaderships from the beginning, throughout the implementation phases, in the decision-making process in order to build a sense of ownership and demand for CB-SDART sustainment as well as set the tone for the key population lay providers;
2. Training and educating key population lay providers to equip them with the skills needed to lead the SDART implementation;
3. Adapting and tailoring SDART to context by developing SOP and revising the SDART’s periphery components in the SOP while the core components remain intact: The original SOP will be developed with the input of the CBO leaderships and staff members during the pre-implementation phase. During the implementation phase, regular meetings will be held to brainstorm and identify gaps in current service and to conclude with a revised version of the SOP where all parties have a mutual agreement;
4. Using evaluative and iterative strategies to assess adherence to SOP: Iterative reflection and evaluation will be conducted internally during the implementation phase. Internal service flow assessment and regular meetings led by the CBO leaderships with the support of IHRI will be conducted to ensure intervention fidelity;
5. Developing stakeholder relationship by engaging external stakeholders who primarily are NHSO and BMA: During the implementation phase, quarterly reports will be submitted to NHSO and BMA to familiarize them with the ongoing implementation at the CBOs. During the post-implementation phase, a strategic meeting with the stakeholders will be conducted to gain public recognition and endorsement for the external sustainability of SDART.

The five strategies described using Proctor’s strategy specification framework [33] are shown in Table 2.

**Table 2:** Implementation strategies and implementation outcome measurement methods

Implementation strategies	Actors	Actions	Action targets	Temporality/Dose	Implementation outcomes	What is measured	Sources of data
Developing stakeholder relationships by engaging CBO leaderships	IHRI	In-depth interviews	CBO leaderships	Once during pre-implementation phase and at months 6 and 12	Sustainability	Attitudes of CBO leadership to CB-SDART service over time The degree to which CBO leadership supports, owns and plans on continuing delivery of CB-SDART	In-depth interviews with CBO leadership as well as staff: Interview notes, audio recordings, transcripts; Meeting minutes from progress update meetings
		Regular meetings for planning and consultation between CBOs and IHRI		Once during pre-implementation phase and at months 1, 3, 6, 9, and 12			
Training and educating KP lay providers	IHRI, PIDCU	Didactic and practical training provided by IHRI and PIDCU; certification; service dry-run	CBO leaderships	Once during pre-implementation phase	Feasibility	Number of CBO providers achieving a passing score on tests Number of CBO providers certified to provide CB-SDART related services The level of competency of CBO providers in leading CB-SDART	Test scores of didactic and practical tests; Certification records; Meeting minutes from coaching sessions
		Ongoing coaching/mentoring provided by IHRI and PIDCU		At months 1, 3, 6, 9, and 12, or upon request			
Adapting and tailoring SDART to CBO context	IHRI, CBO	SOP development	CBO	Once during pre-implementation phase	Feasibility	The degree to which CB-SDART service fits with each CBO The level of comfort in delivering CB-SDART related services The extent to which CB-SDART adaptations are necessary after initial implementation	In-depth interviews: Interview notes, audio recordings, transcripts; Meeting minutes of progress update meetings; SOP revision history records
		In-depth interviews		Once during pre-implementation phase and at months 6 and 12			
		Regular meetings for feedback between CBOs, IHRI, and PIDCU; SOP revision		Once during pre-implementation phase and at months 1, 3, 6, 9, and 12			
Using evaluative and iterative strategies to assess adherence to SOP	IHRI, CBO	Regular meetings for feedback between CBOs, IHRI, and PIDCU; internal service flow assessment	CBO	At months 1, 3, 6, 9, and 12	Fidelity	The degree to which CB-SDART is delivered as intended: Proportion of HIV diagnosed clients who are offered SDART Proportion of clients who accepted SDART which undergoes symptomatic screening Proportion of eligible clients initiating ART within the same day of HIV diagnosis Proportion of clients who are followed up for at least one year to ensure retention in care	eCRF; Service delivery checklists; Meeting minutes of CBO internal meetings; SOP deviation records
Developing stakeholder relationship by engaging external stakeholders	IHRI, CBO	Submission of quarterly progress report	BMA, NHSO	At months 3, 6, 9, and 12	Sustainability	The level of support by NHSO and BMA for CB-SDART The extent of endorsement by NHSO and BMA for CB-SDART	Communication records; Meeting minutes of the strategic meeting
		Strategic meeting with national stakeholders		During post-implementation phase			

*CBO *Community-based Organization, *IHRI *Institute of HIV Research and Innovation, *CB-SDART *Community-based Same-Day Antiretroviral Therapy, *KP *Key Population, *PIDCU *Division of Infectious Diseases, Department of Pediatrics, Faculty of Medicine, Chulalongkorn University, *SOP *Standard Operating Procedure, *SDART *Same-Day Antiretroviral Therapy, *eCRF *Electronic Case Report Form, *ART *Antiretroviral Therapy, *BMA *Bangkok Metropolitan Administration, *NHSO *National Health Security Office

### Service and client outcomes

Relevant service and client outcomes to be assessed using CITS are timeliness, patient centeredness, and function. Timeliness is defined as the time between HIV diagnosis and ART initiation. Patient centeredness is defined as the proportion of ART initiation. Function is defined as the retention in care at months 3, 6, and 12 after ART initiation. Relevant client outcomes to be assessed using quantitative and qualitative methods are satisfaction and symptomology. Satisfaction is defined as the level of satisfaction with CB-SDART among clients using the service. Symptomatology is defined as the virologic suppression (< 50 copies/mL) among clients starting ART at month 6 and month 12.

**Fig. 3:**
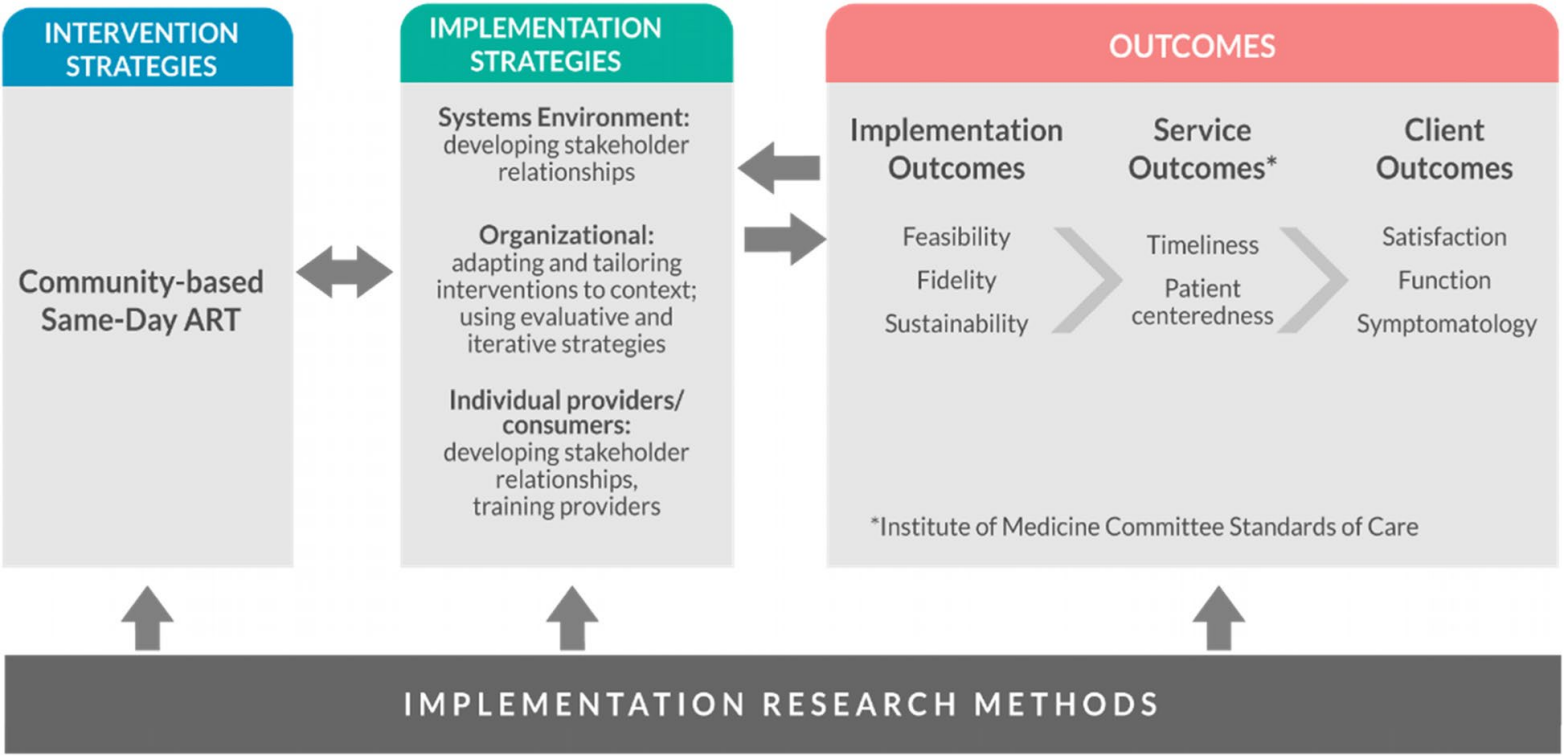
Proctor’s implementation outcome framework applied to community-based same-day antiretroviral therapy implementation study

Implementation, service, and client outcomes relevant to this CB-SDART protocol are summarized in Fig. 3.

### Outcome evaluation

A mixed-method approach will be used to evaluate the implementation, service, and client outcomes. As shown in Table 2, a variety of data sources (i.e., in-depth interviews, meeting minutes, didactic and practical tests, certification records, service delivery checklists, SOP revision history, SOP deviation records, communication records, satisfaction survey, and eCRF) will be used to evaluate the IOs. The eligible stakeholder participants will be invited for regular quantitative and qualitative assessments throughout the pre-, during, and/or post-implementation phases. Mixed-method analysis will be conducted to describe and interpret the findings.

The evaluation of the service and client outcomes will take place during the implementation phase of the study. As shown in Table 3, CITS and mixed-method analysis will be used to analyze data from eCRF, satisfaction survey, and in-depth interviews.

**Table 3:** Evaluation of service and client outcomes

Proctor’s Outcomes	Expected outcomes	What is measured	Data sources	Analysis methods
*Service level:* Timeliness	Eligible clients who are willing and clinically eligible start ART on the same day of HIV diagnosis, and time between diagnosis and ART initiation is significantly reduced compared to before CB-SDART implementation	Days from HIV diagnosis to ART initiation before CB-SDART implementation compared to after CB-SDART implementation Days from HIV diagnosis to ART initiation at CBO 1 (after CB-SDART implementation) compared to at CBO 2 (before CB-SDART implementation during the same period in time)	eCRF: historical routine service data, NAPPLUS Database	CITS
*Service level:* Patient centeredness	CB-SDART initiation is tailored to meet the preferences and needs of clients with high ART initiation uptake among eligible clients	Proportion of clients who initiate ART after being informed about their HIV diagnosis before CB-SDART implementation compared to after CB-SDART implementation Proportion of clients who initiate ART after being informed about their HIV diagnosis at CBO 1 (after CB-SDART implementation) compared to at CBO 2 (before CB-SDART implementation during the same period in time)	eCRF: historical routine service data, NAPPLUS Database	CITS
*Client level:* Satisfaction	High satisfaction with CB-SDART services among clients	Proportion of clients indicating to be satisfied with CB-SDART service	Satisfaction survey	Descriptive analysis
			In-depth interviews: Interview notes, audio recordings, transcripts	Qualitative analysis
*Client level*: Function	High rate of retention in care at three, six, and twelve months after CB-SDART initiation	Proportion of clients retained in care at month 3, month 6, and month 12 after ART initiation, comparing proportions before CB-SDART implementation to after CB-SDART implementation Proportion of clients retained in care at month 3, month 6, and month 12 after ART initiation, comparing proportions at CBO 1 (after CB-SDART implementation) compared to at CBO 2 (before CB-SDART implementation during the same period in time)	eCRF: historical routine service data, NAPPLUS Database	CITS
*Client level:* Symptomatology	High rate of virologic suppression achieved six and twelve months after CB-SDART initiation	Proportion of clients achieving virologic suppression at month 6 and month 12 after ART initiation, comparing proportions before CB-SDART implementation to after CB-SDART implementation Proportion of clients achieving virologic suppression at month 6 and month 12 after ART initiation, comparing proportions at CBO 1 (after CB-SDART implementation) compared to at CBO 2 (before CB-SDART implementation during the same period in time)	eCRF	Descriptive analysis

*ART *Antiretroviral Therapy, *CB-SDART *Community-based Same-Day Antiretroviral Therapy, *CBO *Community-based Organization, *eCRF *Electronic Case Report Form, *NAPPLUS *National AIDS Program Plus, *CITS *Comparative Interrupted Time Series

### Data collection

Various data sources as shown in Tables 2 and 3 will be collected to assess implementation, service, and client outcomes. All data sources, except for the eCRF and satisfaction survey, will be collected by IHRI implementers. Data in the eCRF will be collected by key population lay providers while the satisfaction survey will be self-administered by client participants. Data on AEs will be collected in the database and serious AE will be filed and reported to the IRB by IHRI implementers. Both IHRI implementers and key population lay providers will be trained for standardized data collection. All data will be collected in a secure online database with protected username and password and limited access only to the study team. Personal and medical information of participants that will be collected in this study will be confidential. De-identification processes will ensure that the identity of the participants will be untraceable. Data quality assessment will be conducted at least once every quarter to ensure high-quality dataset by a team of clinical data officers and clinical trial monitor officers from IHRI. Additionally, IHRI will produce a monthly report on CB-SDART service performance for data sharing and cross-check with CBOs and PIDCU.

### Data analysis

Descriptive analysis will summarize demographic characteristics, didactic and practical test scores and certification records (feasibility), adherence to SOP (fidelity), communication record (sustainability), virologic suppression rate (symptomatology), and satisfaction survey (satisfaction) using mean, standard deviation, median, and interquartile range for continuous variables, and frequency and proportion for categorical variables. Qualitative analysis will be conducted to assess feasibility, fidelity, sustainability, and client satisfaction. The recordings of in-depth interviews and meeting minutes will be transcribed and coded using an analytical software or by a team of trained qualitative researchers. The codes will be categorized into meaningful themes deductively according to the research objectives. CITS will be conducted using segmented regression analysis to assess timeliness, patient centeredness, and function. Pre-intervention data will be adjusted to generate a reliable baseline for each study site. The adjusted baseline trend will be used to investigate the impact of CB-SDART in three ways: (1) internal comparison for each CBO before and after CB-SDART launch, (2) external comparison between the implemented CBO and the non-implemented CBO, and (3) the external comparison after all CBOs have implemented CB-SDART. Scatter plots will be used to explore the relationships between time and relevant variables. Analysis of trends, seasonality, and autocorrelation plots will also be conducted to improve the robustness of the analyses [34–36].

### Ethical considerations

This study protocol (version 2.0, dated August 5, 2021) was approved by the Chulalongkorn University Institutional Review Board (IRB No. 437/64). It is registered with Thai Clinical Trial Registry as TCTR20210709004 on July 9, 2021 (http://www.thaiclinicaltrials.org/show/TCTR20210709004). All members of stakeholders (i.e., IHRI, PIDCU, RSAT, SWING, NHSO, and BMA) will be informed about the implementation research. All key stakeholders participating in in-depth interviews and all clients participating in active data collection and/or in-depth interviews will undergo an informed consent process. Informed consent process is waived for secondary data collection. If the protocol is to be modified, the protocol amendment will be submitted to Chulalongkorn University IRB for approval and IHRI will communicate the protocol changes to key and external stakeholders.

## Discussion

To our knowledge, our CB-SDART implementation trial will be the first to generate robust findings at implementation, service, and client levels, and inform possible successful strategies to decentralize and task-shift ART initiation services to trained key population lay providers to care for their own community. Our findings will supplement the emerging evidence that community-based ART initiation may result in better ART uptake, retention, and virologic suppression than the traditional facility-based settings [15], and facilitate the systematic uptake of CB-SDART in other CBOs across Thailand and elsewhere. Additionally, this study will provide the opportunity to strengthen the capacity of key population lay providers in providing HIV care services. This will help facilitate the elevation of the CBOs’ status as healthcare facilities recognized by the Thai national public health agencies and ensure the sustainability of KPLHS and CB-SDART after the study is over. With the new normalcy of the COVID-19 period, we will pioneer the practice of ART initiation via telehealth. This strategy will overcome the geographical and human resource barrier that comes with any disease outbreaks. Ultimately, bringing SDART initiation hubs into the community will potentially accelerate linkage to care, promote service continuation, and curtail the HIV epidemic among key populations in Bangkok.

## Supplementary Information

**Additional file 1.** Overview of study activity schedules.

**Additional file 2.** SPIRIT 2013 Checklist: Recommended items to address in a clinical trial protocol and related documents*.

**Additional file 3.** The TIDieR (Template for Intervention Description and Replication) Checklist^*^.

## Data Availability

Data sharing is not applicable to this article as no datasets were generated or analyzed during the current study. All study materials, including informed consent forms, are in Thai and translations are available upon reasonable request.

## Abbreviations

AE: Adverse event
Anti-HCV: Hepatitis C antibody
ALT: Alanine aminotransferase
ART: Antiretroviral therapy
BMA: Bangkok Metropolitan Administration
CBO: Community-based organization
CB-SDART: Community-based same-day antiretroviral therapy
CFIR: Consolidated Framework for Implementation Research
CITS: Comparative interrupted time series
CT: Chlamydia
EBP: Evidence-based practice
eCRF: Electronic case report form
HBsAg: Hepatitis B surface antigen
IHRI: Institute of HIV Research and Innovation
IO: Implementation outcome
IS: Implementation strategy
KP: Key population
KPLHS: Key population-led health service
LTFU: Lost to follow-up
NG: Gonorrhea
NHSO: National Health Security Office
OI: Opportunistic infection
PEP: Post-exposure prophylaxis
PIDCU: Division of Infectious Diseases, Department of Pediatrics, Faculty of Medicine, Chulalongkorn University
PLHIV: People living with HIV
PrEP: Pre-exposure prophylaxis
RPR: Rapid plasma reagin
RSAT: Rainbow Sky Association of Thailand
SDART: Same-day antiretroviral therapy
SOP: Standard operating procedure
STI: Sexually transmitted infection
SWING: Service Workers In Group Foundation
TB: Tuberculosis
TPHA: Treponema pallidum particle hemagglutination
TRCAC: Thai Red Cross Anonymous Clinic
