# Supplementary material for: Key population-led community-based same-day antiretroviral therapy (CB-SDART) initiation hub in Bangkok, Thailand: a protocol for a hybrid type 3 implementation trial
Source: Implement Sci Commun. 2022 Oct 1;3:101. doi: 10.1186/s43058-022-00352-9 (PMC9526529; doi:10.1186/s43058-022-00352-9)
Supplement: Supplementary file 1 — Additional file 1. Overview of study activity schedules. [file 43058_2022_352_MOESM1_ESM.docx]

Additional file 1. Overview of study activity schedules.

| **Activity** | **Y1**  **Q1: Pre-impl.** | **Y1**  **Q2** | **Y1**  **Q3** | | **Y1**  **Q4** | | **Y2**  **Q1** | | | **Y2**  **Q2** | | | **Y2**  **Q3** | | | **Y2**  **Q4: Post-impl.** | | |
| --- | --- | --- | --- | --- | --- | --- | --- | --- | --- | --- | --- | --- | --- | --- | --- | --- | --- | --- |
| **CBO-1** | | | | | | | | | | | | | | | | | | |
| **Launch of CB-SDART** | |  | |  | |  | | |  | | |  | | |  | | |  |
| **Implementation outcome: Internal Sustainability** | |  | | | | | | | | | | | | | | | | |
| Planning and consultation meeting between CBO, IHRI, and PIDCU | ✓ | ✓ | ✓ | | ✓ | | ✓ | | |  | | |  | | |  | | |
| IDI with CBO leadership and KP lay providers to assess leadership engagement | ✓ |  | ✓ | |  | | ✓ | | |  | | |  | | |  | | |
| **Implementation outcome: External Sustainability** | |  | | | | | | | | | | | | | | | | |
| Quarterly report to BMA and NHSO |  | ✓ | ✓ | | ✓ | | ✓ | | |  | | |  | | |  | | |
| Strategic meeting with BMA and NHSO |  |  |  | |  | |  | | |  | | |  | | | ✓ | | |
| **Implementation Outcome: Feasibility** | |  | | | | | | | | | | | | | | | | |
| Training | ✓ |  |  | |  | |  | | |  | | |  | | |  | | |
| Ongoing coaching and mentoring | ✓ | ✓ | ✓ | | ✓ | | ✓ | | |  | | |  | | |  | | |
| IDI with KP lay providers to assess the fit of CB-SDART at CBO | ✓ |  | ✓ | |  | | ✓ | | |  | | |  | | |  | | |
| Feedback meetings between CBO, IHRI, and PIDCU |  | ✓ | ✓ | | ✓ | | ✓ | | |  | | |  | | |  | | |
| **Implementation outcome: Fidelity** | |  | | | | | | | | | | | | | | | | |
| Internal service evaluation meeting at CBOs |  | ✓ | ✓ | | ✓ | | ✓ | | |  | | |  | | |  | | |
| Service delivery checklist on randomly selected clients |  | ✓ | ✓ | | ✓ | | ✓ | | |  | | |  | | |  | | |
| **Service outcome: Timeliness** | |  | | | | | | | | | | | | | | | | |
| Retrospective collection of # days between HIV diagnosis and ART initiation in the 12 months prior to launch of CB-SDART | ✓ |  |  | |  | |  | | |  | | |  | | |  | | |
| Prospective collection of # days between HIV diagnosis and ART initiation during the 15 months after launch of CB-SDART |  | ✓ | ✓ | | ✓ | | ✓ | | | ✓ | | |  | | |  | | |
| **Service outcome: patient centeredness** | |  | | | | | | | | | | | | | | | | |
| Retrospective collection of % of HIV-positive clients initiating ART in the 12 months prior to launch of CB-SDART | ✓ |  |  | |  | |  | | |  | | |  | | |  | | |
| Prospective collection of % of HIV-positive clients initiating ART during the 15 months after launch of CB-SDART |  | ✓ | ✓ | | ✓ | | ✓ | | | ✓ | | |  | | |  | | |
| **Client outcome: Satisfaction** | |  | | | | | | | | | | | | | | | | |
| Satisfaction exit survey on randomly selected clients |  | ✓ | ✓ | | ✓ | | ✓ | | | ✓ | | |  | | |  | | |
| IDIs with clients to assess satisfaction with CB-SDART |  |  | ✓ | |  | | ✓ | | |  | | |  | | |  | | |
| **Client outcome: Function** | |  | | | | | | | | | | | | | | | | |
| Retrospective collection of % of clients in care at M3, M6, and M12 who were referred by CBO for ART initiation during the 12 months prior to launch of CB-SDART | ✓ | ✓ | ✓ | | ✓ | | ✓ | | |  | | |  | | |  | | |
| Prospective collection of % in care at M3, M6, and M12 among CB-SDART clients who initiated ART during the first 3 months after CB-SDART launch |  |  | ✓ | | ✓ | | ✓ | | | ✓ | | |  | | |  | | |
| **Client outcome: Symptomatology** | |  | | | | | | | | | | | | | | | | |
| Retrospective collection of % of clients who were referred by CBO for ART initiation during the 12 months prior to launch of CB-SDART with VLS at M6 and M12 | ✓ | ✓ | ✓ | | ✓ | | ✓ | | |  | | |  | | |  | | |
| Prospective collection of % of clients with VLS at M6 and M12 after CB-SDART launch |  |  |  | | ✓ | | ✓ | | | ✓ | | |  | | |  | | |
| **CBO-2** | | | | | | | | | | | | | | | | | | |
| **Launch of CB-SDART** | | |  | |  | | |  | | |  | | |  | | |  | |
| **Implementation outcome: Internal Sustainability** | | |  | | | | | | | | | | | | | | | |
| Planning and consultation meeting between CBO, IHRI, and PIDCU | ✓ |  | ✓ | | ✓ | | ✓ | | | ✓ | | |  | | |  | | |
| IDI with CBO leadership and KP lay providers to assess leadership engagement |  | ✓ |  | | ✓ | |  | | | ✓ | | |  | | |  | | |
| **Implementation outcome: External Sustainability** | | |  | | | | | | | | | | | | | | | |
| Quarterly report to BMA and NHSO |  |  | ✓ | | ✓ | | ✓ | | | ✓ | | |  | | |  | | |
| Strategic meeting with BMA and NHSO |  |  |  | |  | |  | | |  | | |  | | | ✓ | | |
| **Implementation Outcome: Feasibility** | | |  | | | | | | | | | | | | | | | |
| Training |  | ✓ |  | |  | |  | | |  | | |  | | |  | | |
| Ongoing coaching and mentoring |  | ✓ | ✓ | | ✓ | | ✓ | | | ✓ | | |  | | |  | | |
| IDI with KP lay providers to assess the fit of CB-SDART at CBO |  | ✓ |  | | ✓ | |  | | | ✓ | | |  | | |  | | |
| Feedback meetings between CBO, IHRI, and PIDCU |  |  | ✓ | | ✓ | | ✓ | | | ✓ | | |  | | |  | | |
| **Implementation outcome: Fidelity** | | |  | | | | | | | | | | | | | | | |
| Internal service evaluation meeting at CBOs |  |  | ✓ | | ✓ | | ✓ | | | ✓ | | |  | | |  | | |
| Service delivery checklist on randomly selected clients |  |  | ✓ | | ✓ | | ✓ | | | ✓ | | |  | | |  | | |
| **Service outcome: Timeliness** | | |  | | | | | | | | | | | | | | | |
| Retrospective collection of # days between HIV diagnosis and ART initiation in the 12 months prior to launch of CB-SDART | ✓ | ✓ |  | |  | |  | | |  | | |  | | |  | | |
| Prospective collection of # days between HIV diagnosis and ART initiation during the 15 months after launch of CB-SDART |  |  | ✓ | | ✓ | | ✓ | | | ✓ | | | ✓ | | |  | | |
| **Service outcome: patient centeredness** | | |  | | | | | | | | | | | | | | | |
| Retrospective collection of % of HIV-positive clients initiating ART in the 12 months prior to launch of CB-SDART | ✓ | ✓ |  | |  | |  | | |  | | |  | | |  | | |
| Prospective collection of % of HIV-positive clients initiating ART during the 15 months after launch of CB-SDART |  |  | ✓ | | ✓ | | ✓ | | | ✓ | | | ✓ | | |  | | |
| **Client outcome: Satisfaction** | | |  | | | | | | | | | | | | | | | |
| Satisfaction exit survey on randomly selected clients |  |  | ✓ | | ✓ | | ✓ | | | ✓ | | | ✓ | | |  | | |
| IDIs with clients to assess satisfaction with CB-SDART |  |  |  | | ✓ | |  | | | ✓ | | |  | | |  | | |
| **Client outcome: Function** | | |  | | | | | | | | | | | | | | | |
| Retrospective collection of % of clients in care at Months 3, 6, and 12 who were referred by CBO for ART initiation during the 12 months prior to launch of CB-SDART | ✓ | ✓ | ✓ | | ✓ | | ✓ | | | ✓ | | |  | | |  | | |
| Prospective collection of % in care at Months 3, 6, and 12 after CB-SDART launch |  |  |  | | ✓ | | ✓ | | | ✓ | | | ✓ | | |  | | |
| **Client outcome: Symptomatology** | | |  | | | | | | | | | | | | | | | |
| Retrospective collection of % of clients who were referred by CBO for ART initiation during the 12 months prior to launch of CB-SDART with VLS at Months 6 and 12 | ✓ | ✓ | ✓ | | ✓ | | ✓ | | | ✓ | | |  | | |  | | |
| Prospective collection of % of clients with VLS at Months 6 and 12 after CB-SDART launch |  |  |  | |  | | ✓ | | | ✓ | | | ✓ | | |  | | |

Abbreviations – Y: year, Q: quarter, ART: antiretroviral therapy, BMA: Bangkok Metropolitan Administration, CBO: community-based organization, CB-SDART: community based same day ART, IDI: in-depth interview, IHRI: Institute for HIV Research and Innovation, PIDCU: Division of Infectious Diseases, Department of Pediatrics, Faculty of Medicine, Chulalongkorn University, NHSO: National Health Security Office, VLS: viral load suppression
